# Editorial: Real estate in developing economies: lens of public health economics and interdisciplinary health sciences

**DOI:** 10.3389/fpubh.2023.1267518

**Published:** 2023-09-12

**Authors:** Hui Jin, Fu-sheng Tsai

**Affiliations:** ^1^School of Economics and Management, Zhanjiang Sci-Tech University, Hangzhou, China; ^2^Department of Business Administration, Cheng Shiu University, Kaohsiung, Taiwan; ^3^Center for Environmental Toxin and Emerging-Contaminant Research, Cheng Shiu University, Kaohsiung, Taiwan; ^4^Super Micro Mass Research and Technology Center, Cheng Shiu University, Kaohsiung, Taiwan

**Keywords:** real estate, health economics, public health, developing economies, environment

## Introduction

Real estate markets have suffered from high vulnerability in recent decades. Especially in emerging countries, real estate markets are rapidly moving in either promisingly positive or sadly negative directions. Facing the paradoxical development of real estate industries and their important impact on our society, we need more integrative research on real estate phenomena for complex problems, especially those highly related to society-wide wellness, health, economic problems, and so on. For example, we have eye-witnessed that public health problems have crashed the economy and real estate market during major public health crises such as the COVID-19 pandemic, especially for those developing countries with vulnerable economic systems (1).

Even though the number of interdisciplinary research on the real estate market and public health is growing, more studies are needed for fine-grained knowledge accumulation and advancement. For example, a major public health crisis will cause health problems and great uncertainty, and then impact the consumption and economic growth of a country. In another instance, in those developing countries where the real estate industry is gradually becoming a pillar industry, what kind of impact will real estate have on the economy and public health? Therefore, interdisciplinary research on real estate markets and public health is helpful to explore and clarify some complex phenomena and problems. Furthermore, this interdisciplinary research may help innovative lines of literature both in the field of public health and real estate. Existing research has noticed some relations between real estate and public health, but has not systematically studied the related problems and phenomena.

The articles in this Research Topic not only represent the new thoughts of real estate from a special lens of public health economics and interdisciplinary health sciences but also include some social science problems closely related to the two core topics (real estate and public health), such as building work, mental health, COVID-19, urban development, the environment, and so on. In this editorial, we briefly review and critique these articles, in order to stimulate more reflections for future studies.

## Research frontiers and imagination for future studies

This section respectively and broadly reviews the seven articles in this Research Topic. Jin et al. point out that, with the large-scale demolition of urban villages in China, the supply of affordable housing for migrant workers has decreased a lot, which may lead to a large outflow of migrant workers and then impact the sustainable development of urban areas. Thus, they focus on the relationship between urban village demolition and the migration decisions of migrant workers in China. Their results show that the large-scale demolition of urban villages has significantly impacted the decisions of migrant workers. Additionally, life satisfaction and rent price are factors that significantly affect migrant workers' decision to leave or stay in the city. This article contributes to exploring the relationship between large-scale urban village demolition and the migratory decision (leave or stay in the city) of migrant workers, so as to provide theoretical and practical references for urban village redevelopment policies.

Tang et al. have done an interesting job in exploring a potential and useful conceptual model to analyze whether sustainable transformational leadership supported sustainable innovation ambidexterity in green building industries. Further, they also examine the relationship between sustainable transformational leadership and sustainable innovation ambidexterity via the mediating effect of psychological capital. This article contributes to the existing knowledge that employees with sustainable transformational leadership exhibit high psychological capital, perceived organizational support, and sustainable innovation ambidexterity in green building industries.

Liang et al. state that COVID-19 has had a great impact on the global economy, especially on the tourism industry because of the lockdown policies, disease, and uncertainty during the pandemic. They focus on the cultural tourism industry during the COVID-19 pandemic and build a theoretical framework for the renewal and development of urban historical blocks and humanism in urban development. This article gives a comprehensive analysis of the development of the Qingming Bridge Historical and Cultural Block during the pandemic. This study could be seen as a first step and it may inspire future studies to empirically examine the relationships between major public health crises and the cultural tourism industry.

Shuying and Xuedong work creatively on the topic of “Evaluation of Mexican poverty reduction policies under the COVID-19 pandemic impacts.” They find that the Mexican government implemented social relief policies that adhered to the principle of “for the good of all, first the poor (Por el bien de todos, Primero los pobres),” but neither could the decrease in income be avoided nor the level of poverty reduced during the pandemic. The relief policies reduced the support for the low-income population and further aggravated the deterioration of poverty because of their indifferent application with respect to the high-income and low-income populations. This paper reveals that the Mexican government's policies to reduce income inequality did not work during the COVID-19 pandemic, and then analyzes the deep political and economic reasons. It provides policy reference for countries with widening income gaps.

Ma et al. provide a public health policy analysis of the Healthy Cities Pilot program and the management of municipal solid waste in China. They present an interesting result as the collection amount and harmless treatment capacity increased by 15.66 and 10.75%, respectively, after the cities were selected as part of the Healthy Cities Pilot. Furthermore, the Healthy Cities Pilot program had a stronger impact on the management of municipal solid waste in the cities with higher administrative levels and larger sizes. This article provides a reference for theoretical research and practical formulation of public health policies.

Chen and Tang present interesting research on the green building industry and health from a micro perspective. The results show that psychological capital has a significant effect on green innovation ambidexterity, subjective health, and psychological health; furthermore, green innovation ambidexterity has a substantial impact on green performance in the green building industry. Furthermore, both subjective health and psychological health significantly affect individuals' organizational performance. This article not only focuses on the new concept of “green building,” but also represents an interdisciplinary study in real estate and health.

Another interesting and important work, done by Yang et al., has gives us a lot to think about regarding a broad academic landscape. They study the impact of environmental regulation on public health since implementing environmental regulation policy is a meaningful way to control environmental pollution and then to impact public health. The result shows that the Two Control Zones policy (an environmental regulation policy in China) significantly improved the public health situation, and reduced the incidence rate of respiratory diseases in Two Control Zones areas by 5.7%. Further, this positive effect was greater in non-provincial capital cities than in capital cities. This study contributes to filling the research gap between environmental regulation policy and public health in developing economies.

## Concluding remarks

According to the important criteria and lines from the Frontiers' (publisher's) web page (https://www.frontiersin.org/about/research-topics) about Research Topics, which stresses that the GE of a Research Topic should “unite the world's leading experts around the hottest topics, stimulating collaboration and accelerating science,” we find that there is more to do, even with the hard work that has been done by all authors of these seven papers in this Research Topic. We tried our best to attract interesting and thought-stimulating articles from all over the world, though, other similar Research Topics may do better in encouraging research in this field.

And if there is a group of well-known and cutting-edge scholars in the field involved, this can be done better, which is conducive to intergenerational knowledge transfer and co-creation. In fact, interdisciplinary collaborative research from different research groups and different career stages matters a lot, because they share similar knowledge bases but also possess heterogeneous research interests, problems, skills, and resources, which may create better research. A Research Topic could be seen as a virtual platform that can effectively collect, review, and edit. It provides readers with a one-stop knowledge and research storage. However, the process of forming such a Research Topic requires a significant amount of time expenditure. We sincerely hope that this Research Topic is only the first step toward our goal of “accelerating science.” There is still a lot of work that needs to be done, and the next crucial step is the interdisciplinary cooperation of researchers from different fields.

## Author contributions

HJ: Writing—original draft, Writing—review and editing. F-sT: Writing—review and editing.
